# Suprasellar Meningiomas: An Experience of Four Cases With Brief Review of Literature

**DOI:** 10.7759/cureus.12470

**Published:** 2021-01-04

**Authors:** Saad Khalil Chaudhry, Rabail Raza, Muhammed A Naveed, Iffat Rehman

**Affiliations:** 1 Radiology, Shaukat Khanum Memorial Cancer Hospital and Research Centre, Lahore, PAK; 2 Radiology, Shaukat Khanum Hospital, Lahore, PAK

**Keywords:** meningioma, suprasellar tumor, pituitary adenoma, neurosurgery

## Abstract

Suprasellar meningiomas make a relevant differential when it comes to sellar/suprasellar masses. The most common pathology in this location is pituitary adenomas. It is imperative to differentiate the two entities based on imaging as the clinical picture, and sometimes the biochemical profile can show significant overlap. It is also essential for the neurosurgeons to have a preoperative diagnosis as the behavior of both tumors is different. This piece will give a pictorial review of the imaging features of suprasellar meningiomas, in patients who presented to us with sellar/suprasellar masses. The aim is to help the radiologists as well as fellow clinicians to diagnose this entity with confidence based on imaging.

## Introduction

Meningioma accounts for 15-25% of all intracranial tumors [1,2], with an incidence of five cases per 100,000 persons per year [3]. Meningiomas are common at the skull vault, skull base, parasagittal location, sphenoid ridge, cavernous sinus, and cerebral convexity [4]. Sellar-based tumors comprise 15% of all intracranial masses [5]. The most common sellar and suprasellar masses are the pituitary adenomas, accounting for almost 90% of the cases [6]. Other sellar-based tumors include craniopharyngiomas that can occur anywhere along the infundibulum (from the floor of the third ventricle to the pituitary gland). Meningiomas themselves constitute only 1% of the sellar masses [7]. Suprasellar/parasellar meningiomas occur in 5-10% of all intracranial meningiomas [8]. Meningiomas arising from the diaphragmatic sellae or sellae turcica are seldom seen (1%) [7]. The most common presenting complaints are a visual disturbance and headache [9]. Endocrine abnormalities like hyperprolactinemia are also observed, in as many as 36% of the cases [7]. It can be impossible to clinically differentiate other sellar masses from a sellar meningioma based on symptoms as there is considerable overlap.

Radiologically contrast-enhanced magnetic resonance imaging plays a defining role in evaluating the extent of a tumor. It gives accurate guidance to and within the sella, provides precise information about the extent of resection, and alert the surgeon to the development of complications.

Meningiomas are generally highly vascular tumors that obtain their blood supply from the arteries of the adjacent dura and bone, therefore, there is a high risk of intraoperative bleeding [10]. In these cases, preoperative endovascular embolization is suggested, where interventional neuroradiology now allows super-selective catheterization of the arteries that supply a tumor. It should be noted that only meningiomas taking their supply from external carotid artery branches are embolized, while the internal carotid artery (ICA) supply is spared in these embolization procedures.

## Case presentation

Case 1

**Figure 1:**
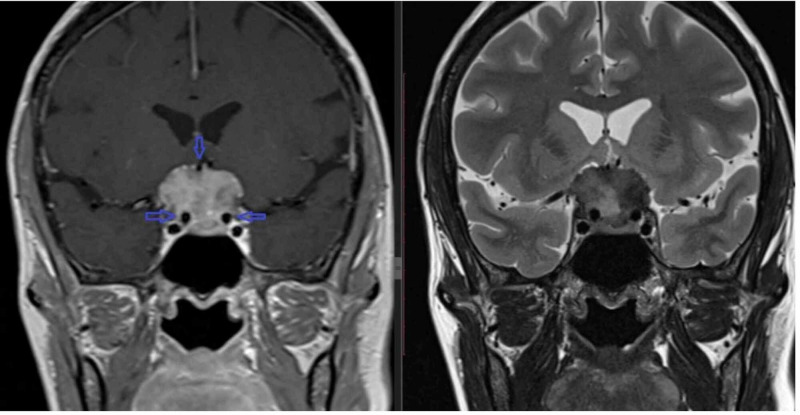
T1-weighted post-contrast shows intensely enhancing sellar and suprasellar mass, with encasement of the supraclinoid part of bilateral ICA (blue arrows). There is the encasement of the right anterior cerebral artery (A1 segment). T2-weighted coronal sequences show mass lesion returning isointense signals similar to gray matter. ICA: internal carotid artery

The first patient was a 54-year-old female who presented with a chief complaint of headache that worsened over time. The pain was relieved by pain killers temporarily. There were also complaints of decreased vision, more in the right eye, which worsened over time. On examination, there was an almost complete loss of right temporal field vision. No visual field loss was identified on the left side. Her hormonal profile was unremarkable apart from suppressed thyroid-stimulating hormone (TSH). However, there were no clinical signs of thyroid overactivity. A thyroid scan was performed, which showed a hyperfunctioning multinodular goiter. Initial MRI was a non-targeted study, raising suspicion of pituitary macroadenoma. A repeat targeted MRI at our hospital showed a large sellar/suprasellar mass involving the tuberculum sellae. The tumor extended anteriorly over the planum sphenoidale. Posteriorly, the tumor involved the pituitary fossa compressing the normal pituitary, and the interpeduncular cistern. There was a significant mass effect and displacement of optic chiasm superiorly. MRI signals were isointense to gray matter on T1- and T2-weighted sequences, with intense homogenous postcontrast enhancement, suggestive of meningioma. The tumor was encasing bilateral internal carotid arteries (Figure 1). The initial multidisciplinary team planned for surgical resection, but the patient refused surgery as there was a significant risk of vascular injury, stroke, and even death, eventually the patient was referred for radiation to a clinical oncologist.

Case 2

**Figure 2:**
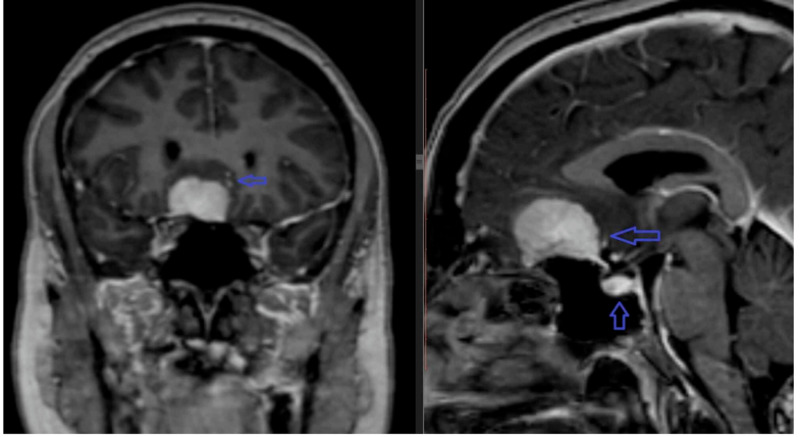
Non-targeted MRI (coronal and sagittal post-contrast images) show an intensely enhancing extra-axial mass at planum sphenoidale with a dural tail (seen on the sagittal sequence, blue arrows). Also note that normal-appearing pituitary in the sella. Note: The TS, DS, and planum sphenoidale meningiomas are grouped as one entity in most publications. TS: tuberculum sellae; DS: diaphragma sellae

The second patient was a 47-year-old female. She was a known case of metastatic ovarian cancer and had a PET-CT scan for staging purposes. The patient had an undocumented episode of changes in behavior, headaches, and seizures which raised concerns for possible intracranial metastatic disease. MRI brain hence was performed for further evaluation. MRI showed an avidly enhancing extra-axial soft tissue mass at the planum sphenoidale, with dural tail sign posteriorly (Figure 2). The anterior cerebral arteries were displaced towards the left, with an associated mild midline shift. There was no significant mass effect on the optic chiasm, as the mass was predominantly anterior to the chiasmatic sulcus. The tumor returned similar signal intensity to that of cortex on T1- and T2-weighted sequences, without sellar extension. The patient’s symptoms improved on anticonvulsant medication. No active intervention was advised.

Case 3

**Figure 3:**
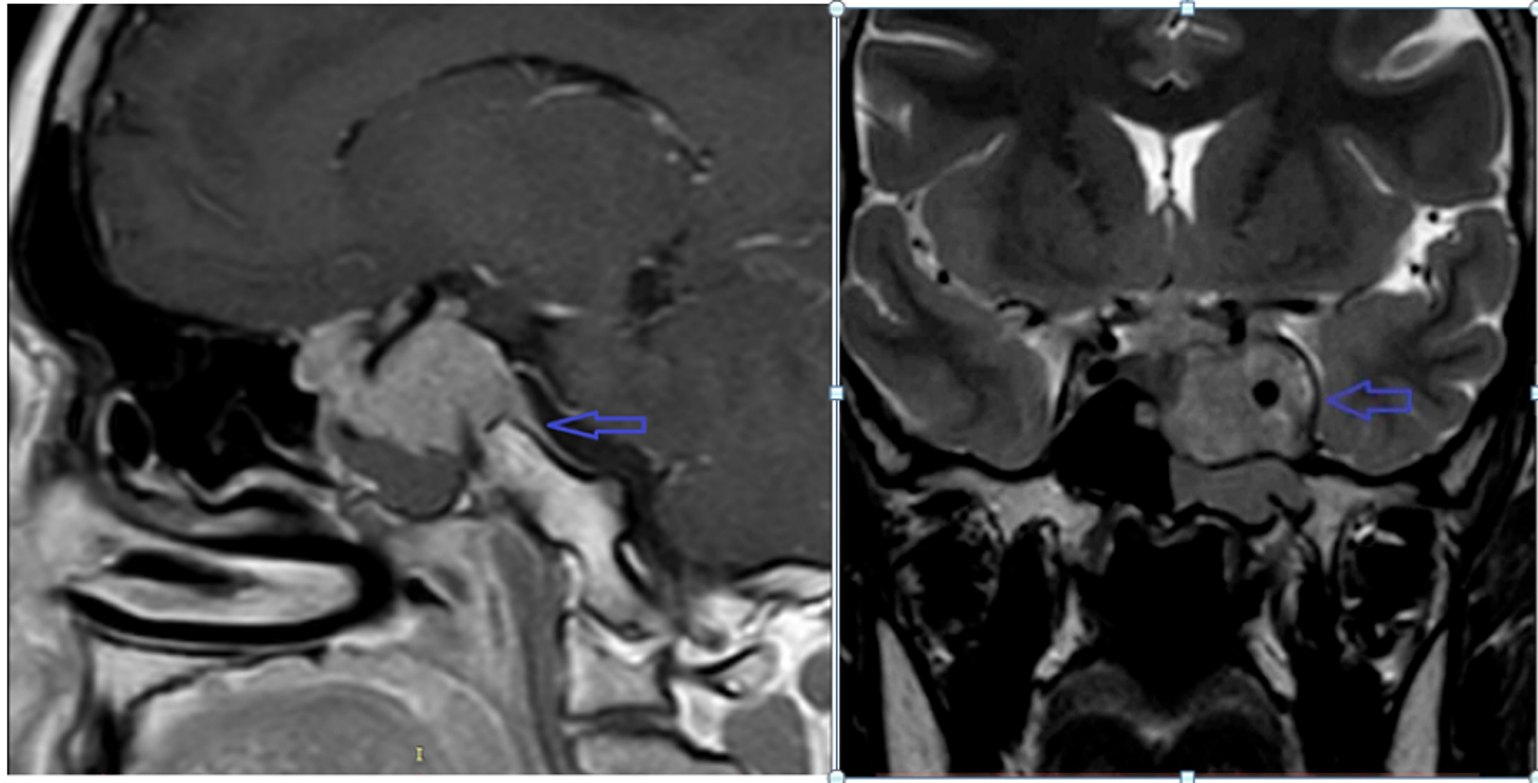
Post-contrast T1 and coronal T2-weighted sequences show a large lobulated parasellar and sellar mass showing avid enhancement. There is the encasement of the left internal carotid artery, with dural tail along the left lateral aspect. Also note the enhancing mass involving the optic chiasm (blue arrows).

The third patient was a 27-year-old female. She was married for 12 years with primary infertility. She was on hormonal therapy with associated galactorrhea. She also complained of headache and decreased vision of the left eye. Her hormonal profile showed markedly raised prolactin levels (10,000 ng/ml), which raised suspicion of pituitary adenoma. MRI showed an avidly enhancing soft tissue in the left parasellar and sellar region, with the involvement of the pituitary fossa. The pituitary had homogenous enhancement and was compressed along the right sellar wall, and the pituitary stalk displaced towards the right. The dural tail was along the left lateral aspect of the tumor, with the involvement of the left internal carotid artery, and partial encasement of the left anterior cerebral artery. It had a mild mass effect and encasement on the left optic nerve and optic chiasm (Figure 3). She got referred to an outside hospital facility for further management.

Case 4

**Figure 4:**
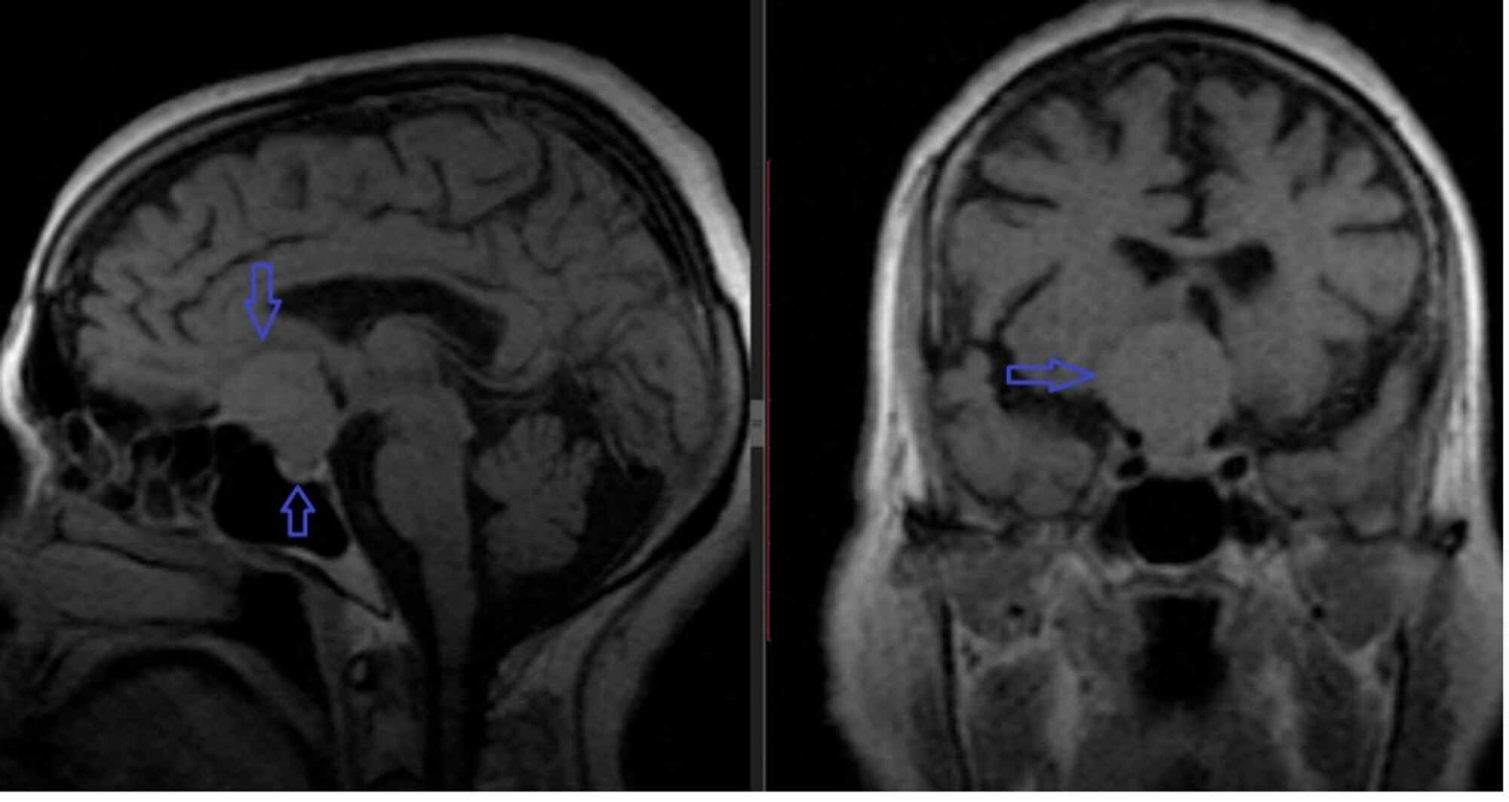
Sagittal and coronal T1-weighted images show a suprasellar mass at the tuberculum sellae, slightly to the right of midline. The mass is compressing the pituitary gland with compression of the optic chiasm superiorly (blue arrows).

The fourth patient who had a diagnostic scan performed elsewhere reported at our institute was a 70-year-old female with a complaint of right eye loss of vision. MRI performed, showed a suprasellar enhancing tumor at the tuberculum sellae. There was compression of the pituitary gland showing uniform enhancement. There was encasement of the right anterior cerebral artery with the involvement of the optic chiasm (Figure 4).

## Discussion

Meningiomas originate from the meningocytes and arachnoid cap cells [7]. MRI is the modality of choice for evaluating the suprasellar masses, due to its superior spatial resolution and multiplane imaging technique [10]. Meningiomas in the suprasellar and parasellar region are not uncommon. However, for the radiologist, it is important to differentiate suprasellar meningiomas from other sellar and suprasellar masses, including the more common pituitary adenomas and craniopharyngioma, as it affects the management of the patient. Another role of the radiologist is to describe the local extension of the tumor and encasement of major vessels, particularly the internal carotid artery or the anterior cerebral artery [11], guiding the neurosurgeons about the possible approach and post-surgical outcomes.

Meningiomas in suprasellar and parasellar regions can be further divided depending on their site of origin, planum sphenoidale, tuberculum sellae, or diaphragmatic sellae. These tumors are common in females with a female to male ratio of 6:1. The mean age documented in the literature is 52 years (median 50, range 30-78), our cases also fall in this age category [8].

Meningiomas are extra-axial masses, most of them display all the imaging features of an extra-axial tumor, including CSF cleft, displacement, and mass effect on the underlying brain cortex [12].

Meningiomas, like suprasellar macroadenoma, are often isointense to cortical gray matter on T1-weighted images [13]. On T2-weighted sequences, adenomas are usually heterogeneous, whereas meningiomas are isointense-hyperintense to gray matter. The presence of calcification can alter the signal characteristics, giving a predominantly hypointense signal [10]. Rarely, atypical features like cystic components or hemorrhage are observed. One of the features specific for meningiomas is the presence of hyperostosis, a focally thickened bone, which is best appreciated on CT images, unfortunately, CT scan images were not available to illustrate findings, MRI, however, did not show significant osseous sclerosis in our cases [10]. Contrast enhancement in meningiomas is almost always brisk, secondary to its highly vascular nature, and homogenous [14]. Pituitary macroadenoma, on the other hand, can have variable enhancement. Meningiomas often demonstrate linear enhancement along the dura, giving the classical “dural tail sign” [10]. The dural tail sign is quite specific but not exclusive to meningiomas.

Pre-surgical diagnosis of meningiomas is imperative because of their fibrous content and expression of angiogenesis factors, making them tenacious and exceptionally vascular. It can lead to significant intra and perioperative morbidity and mortality secondary to hemorrhage compared with pituitary adenomas [15]. Although the aim of surgery is total excision of the tumor, but that is rarely possible due to the mentioned factors [12]. Radiotherapy can be offered as an alternative or as adjuvant therapy to surgery [16]. Stereotactic radiotherapy has better results and a lower rate of complications compared to conventional radiotherapy.

## Conclusions

Sellar and suprasellar meningiomas represent a significant group of intracranial tumors. The surgical treatment presents several difficulties as they are well known to involve critical neurovascular structures. Because meningiomas are so common, the radiologist must be aware of their less frequent and uncharacteristic imaging features to suggest the correct diagnosis in atypical cases.

## References

[1] Amirjamshidi A, Mortazavi SA, Shirani M, Saeedinia S, Hanif H (2017). Coexisting pituitary adenoma and suprasellar meningioma—a coincidence or causation effect: report of two cases and review of the literature. J Surg Case Rep.

[2] Furtado SV, Venkatesh PK, Ghosal N, Hegde AS (2010). Coexisting intracranial tumors with pituitary adenomas: genetic association or coincidence?. J Cancer Res Ther.

[3] Longstreth WT, Dennis LK, McGuire VM, Drangsholt MT, Koepsell TD (1993). Epidemiology of intracranial meningioma. Cancer.

[4] Rohringer M, Sutherland GR, Louw DF, Sima AAF (1989). Incidence and clinicopathological features of meningioma. J Neurosurg.

[5] Terada T, Kovacs K, Stefaneanu L, Horvath E (1995). Incidence, pathology, and recurrence of pituitary adenomas: study of 647 unselected surgical cases. Endocr Pathol.

[6] Valassi E, Biller BMK, Klibanski A, Swearingen B (2010). Clinical features of nonpituitary sellar lesions in a large surgical series. Clin Endocrinol.

[7] Kwancharoen R, Blitz AM, Tavares F, Caturegli P, Gallia GL, Salvatori R (2014). Clinical features of sellar and suprasellar meningiomas. Pituitary.

[8] Ajlan AM, Choudhri O, Hwang P, Harsh G (2015). Meningiomas of the tuberculum and diaphragma sellae. J Neurol Surg B Skull Base.

[9] Sathananthan M, Sathananthan A, Scheithauer BW, Giannini C, Meyer FB, Atkinson JL, Erickson D (2013). Sellar meningiomas: an endocrinologic perspective. Pituitary.

[10] Johnsen DE, Woodruff WW, Allen IS, Cera PJ, Funkhouser GR, Coleman LL (1991). MR imaging of the sellar and juxtasellar regions. RadioGraphics.

[11] Ciric I, Rosenblatt S (2001). Suprasellar meningiomas. Neurosurgery.

[12] Whittle IR, Smith C, Navoo P, Collie D (2004). Meningiomas. Lancet.

[13] FitzPatrick M, Tartaglino LM, Hollander MD, Zimmerman RA, Flanders AE (1999). Imaging of sellar and parasellar pathology. Radiol Clin North Am.

[14] Donovan JL, Nesbit GM (1996). Distinction of masses involving the sella and suprasellar space: specificity of imaging features. AJR Am J Roentgenol.

[15] Sheehan MT, Atkinson JL, Kasperbauer JL, Erickson BJ, Nippoldt TB (1999). Preliminary comparison of the endoscopic transnasal vs the sublabial transseptal approach for clinically nonfunctioning pituitary macroadenomas. Mayo Clin Proc.

[16] Soyuer S, Chang EL, Selek U, Shi W, Maor MH, DeMonte F (2004). Radiotherapy after surgery for benign cerebral meningioma. Radiother Oncol.

